# Self-Assembly Fluorescent Cationic Cellulose Nanocomplex via Electrostatic Interaction for the Detection of Fe^3+^ Ions

**DOI:** 10.3390/nano9020279

**Published:** 2019-02-16

**Authors:** Haoying Wang, Xiu Ye, Jinping Zhou

**Affiliations:** Department of Chemistry, Wuhan University, Wuhan 430072, China; wang159hy@gmail.com (H.W.); yx444131997@gmail.com (X.Y.)

**Keywords:** aggregation-induced emission, quaternized cellulose, electrostatic interaction, fluorescence, Fe^3+^ ions

## Abstract

In this work, an aggregation-induced emission (AIE) sensor for the detection of Fe^3+^ ions was fabricated through the electrostatic interaction between 1,1,2-triphenyl-2-[4-(3-sulfonatopropoxyl)-phenyl]-ethene sodium salt (SPOTPE) and quaternized cellulose (QC). The structure and properties of the SPOTPE/QC nanocomplex were studied by using ^1^H NMR, spectrofluorophotometer, transmission electron microscopy (TEM), and dynamic laser light scattering (DLS). An aqueous solution of SPOTPE and QC resulted in a remarkably enhanced cyan fluorescence in comparison to that of the SPOTPE solution. Strong through-space electrostatic interaction between SPOTPE and QC is the main cause for the fluorescence emerging. The fluorescence of the SPOTPE/QC solutions show good stability over a wide pH range of 5.0–10.0. When introducing Fe^3+^ ions into the SPOTPE/QC solution, the fluorescence quenched within 5 s. SPOTPE/QC solutions exhibited high selectivity and sensitivity for the detection of Fe^3+^ ions with ignored interferences from other ions, and the detection limit was determined to be 2.92 × 10^−6^ M. The quenching mechanism was confirmed to be the consequence of the binding interactions between Fe^3+^ ions and SPOTPE/QC complex.

## 1. Introduction

Fe^3+^ is one of the most abundant and common metal ions and plays an important role in many fields, such as in the biological system, environment, and industrial areas [1]. As one of the essential fundamental elements, Fe^3+^ has an important relationship with biological processes, including oxygen metabolism, electron transfer, and transcriptional regulation [2,3]. When the amount of Fe^3+^ is excessive or insufficient, it will cause serious problems to an organism. Many notorious human diseases, such as Alzheimer’s and Parkinson’s diseases [4], are related to Fe^3+^. Moreover, Fe^3+^ is also involved in many natural phenomena, such as the synthesis of chlorophyll, the reduction of nitrate, and the metabolism of oxygen [5]. In addition, Fe^3+^ is a crucial factor affecting the growth of phytoplankton in water [6]. Up to now, some quantitative and qualitative methods, such as inductively coupled plasma-mass spectrometry, atomic absorption spectrometry, electrochemical stripping analysis, spectrophotometry, colorimetry, and fluorescent approaches, have been constructed [6,7]. Most of the methods are either operated with relatively complex and expensive instruments, or difficult to implement and time-consuming. Therefore, an easy-operation, quick-response, and visual analysis approach for Fe^3+^ detection remains a great necessity. 

Fluorescent techniques have attracted increasing interest for their easy-operation, biocompatibility and cost-effective properties [8]. Many types of fluorescent probe have been designed based on two common phenomena [9,10], which are aggregation-caused quenching (ACQ) and aggregation-induced emission (AIE) [11]. Since the AIE phenomenon was proposed in 2001 [12], it has promoted technological innovations and insights into some working mechanisms and physical properties [13] of AIE molecules, such as the restriction of intramolecular motions for AIE behavior, and the process of electrostatic attraction in the detection field [13,14]. Furthermore, AIE molecules can be utilized to design probes for chemosensor and bioimaging due to their specific fluorescent properties [15,16]. For example, some probes have been designed to achieve the detection of analytes by “turning-on” the fluorescence [17,18,19], while others were constructed based on the AIE effect, and the fluorescence could be quenched by the analytes [20]. Apart from the detection of analytes, some other applications have been provided, such as the detection of pH, radiation, temperature, and bioimaging [21,22,23]. Zhou et al. reported a tetraphenylethene (TPE)-containing poly(acrylate) for the detection of nitro compounds [24]. Liu et al. fabricated a fluorescent probe by combining a silole compound and a polyelectrolyte for the detection of gamma-ray radiation [21]. Li et al. synthesized a series of temperature sensitive polymers with a TPE structure [25]. Wang et al. prepared a fluorescent probe by attaching TPE to the backbone of chitosan, which could enable cell tracing for as long as 15 passages [26]. 

TPE is a typical AIE molecule used mostly for detection, however it cannot dissolve in aqueous solutions, which limits its application. One way to deal with this issue is to attach TPE onto the water-soluble polymer in order to operate in the aqueous environment [27]. Niu et al. conjugated TPE in a block polyurethane copolymer, which can assemble into micelles in water and could be used in bioimaging [27]. Li et al. constructed a fluorescent probe from charge-generation polymers and a TPE derivative, and the probe was well-dissolved in water and showed sensitivity to the presence of H_2_O_2_ or thiols [28]. Though the chemical reaction between water-soluble polymers and TPE molecules make them possible for applicating in water, the approaches are inefficient and difficult to operate [29,30,31]. In this work, we constructed a fluorescence nanocomplex by introducing 1,1,2-triphenyl-2-[4-(3-sulfonatopropoxyl)-phenyl]-ethene sodium salt (SPOTPE) into the quaternized cellulose (QC) aqueous solutions. SPOTPE and QC attracted each other for the electrostatic interactions between -SO_3_^−^ groups of SPOTPE and -(CH_3_)_3_N^+^ groups of QC. The obtained SPOTPE/QC solutions presented a remarkable cyan fluorescence due to the aggregation of SPOTPE molecules [32,33]. Moreover, the SPOTPE/QC solutions demonstrated a selective and sensitive detection to Fe^3+^ in the absence/presence of interfering metal ions, indicating potential applications in the field of chemosensing.

## 2. Materials and Methods

### 2.1. Materials

QC with a degree of substitution (DS) of 0.52 was synthesized according to a previous work [34]. Bromotriphenylethylene, 4-hydroxyphenylboronic acid, tetrakis(triphenyl-phosphine) palladium(0), and tetrabutyl ammonium bromide (TBAB) were purchased from Aladin Ltd. (Shanghai, China) and used as received. The other reagents of analytical grade were supplied by Sinopharm Chemical Reagent (Shanghai, China), and were used without further purification. Ultrapure water was used throughout the experiment. 

### 2.2. Synthesis of SPOTPE

SPOTPE was synthesized according to the references [35,36] (Scheme S1). Bromotriphenylethylene (4.00 g, 11.93 mmol), 4-hydroxyphenylboronic acid (1.97 g, 14.28 mmol), potassium carbonate aqueous solution (2 mol/L, 7.0 mL), tetrabutyl ammonium bromide (150 mg), and Pd(PPh_3_)_4_ (25 mg) were dissolved into 50 mL degassed tetrahydrofuran in a three-necked flask equipped with a magnetic stirrer under nitrogen atmosphere. The mixture was heated to reflux and stirred for 24 h at 60 °C, and then cooled to room temperature and extracted with ethyl acetate. The organic layer was purified by column chromatography on silica gel using petroleum ether/acetone (v/v = 10:1) as eluent to obtain 1,1,2-triphenyl-2-(p-hydroxyphenyl)-ethene (TPEOH). 

TPEOH (0.35 g, 1.00 mmol) was dissolved in 14 mL anhydrous ethanol with dropwise adding the NaOEt (0.07 g, 1.05 mmol) in 7 mL anhydrous ethanol. The reaction was conducted in a 100 mL round-bottom flask, which was equipped with a magnetic stirrer under nitrogen atmosphere. After stirring under nitrogen atmosphere for 1 h at 25 °C, 1,3-propanesultone (0.13 g) in 7.5 mL ethanol was added. The mixture was stirred for another 12 h under nitrogen atmosphere, and then the product was obtained by filtration. The solid was purified through washing with acetone and ethanol thrice alternately, and vacuum dried to collect a white powder of SPOTPE. 

The chemical structures of TPEOH and SPOTPE were identified from ^1^H NMR spectra (Figure S1, 300 MHz, *d*_6_-DMSO, δ): TPEOH, 9.37 (s, 1H); 7.16–6.91 (m, 15H); 6.75–6.72 (d, 2H); 6.52–6.48 (d, 2H). SPOTPE, 7.17–6.96 (m, 15H); 6.85–6.81 (d, 2H); 6.69–6.65 (d, 2H); 4.01–3.90 (t, 6H). 

### 2.3. Preparation of SPOTPE/QC Solutions

SPOTPE and QC were dissolved in ultrapure water to obtain the stock solutions with concentrations of 2.0 mg/mL, which were stored at room temperature before use. A 5 μL SPOTPE solution was added to the 100 μL QC solution, and the mixture was diluted to 1 mL with ultrapure water. The final concentrations of SPOTPE and QC in the SPOTPE/QC solution were 0.01 mg/mL and 0.20 mg/mL, respectively. 

### 2.4. Fluorescence Detection of Fe^3+^ Ions

For the detection of Fe^3+^, an increasing amount of Fe^3+^ was added to the as-prepared SPOTPE/QC solutions, and then the fluorescence spectra of the solutions were monitored with the spectrofluorophotometer (Perkin Elmer LS55, PerkinElmer Ltd., BUCKS, UK). To verify the selective detection of Fe^3+^, 300 μM of the interfering ions (M^n+^: K^+^, Li^+^, Na^+^, Ag^+^, NH_4_^+^, Pb^2+^, Zn^2+^, Mg^2+^, Ba^2+^, Ca^2+^, Cd^2+^, Co^2+^, Cu^2+^, Fe^2+^, Cr^3+^ or Al^3+^) were added to the SPOTPE/QC solution with/without Fe^3+^ ions, and then they were monitored by the spectrofluorophotometer.

### 2.5. In Vitro Release of SPOTPE from SPOTPE/QC Complexes with/without Fe^3+^ Ions

The SPOTPE/QC solutions with/without Fe^3+^ ions were prepared according to the method mentioned above (*c*_QC_ = 0.2 mg/mL, *c*_SPOTPE_ = 0.01 mg/mL, and *c*_Fe_^3+^ = 300 μM). 20 mL of the solutions were placed into the dialysis tubes (*M*_w_ cutoff 8000 Da), and dialyzed against 50 mL ultrapure water at 25 °C with continuous shaking at 150 rpm. At predetermined intervals, the external release medium was taken out for measurements and was replaced by the fresh ultrapure water. The amounts of SPOTPE released from the complex solutions were recorded by UV-Vis spectrophotometer (UV-6, Mapada, Shanghai, China) at 310 nm (the maximum adsorption peak of SPOTPE solution). The cumulative SPOTPE release was calculated according to the following equation:

$$\mathrm{Cumulative}\;\mathrm{release}\;\left(\%\right)=\frac{W_{t}}{W_{SPOTPE}}\times 100$$

where W*_t_* represents the weight of SPOTPE released from the complex solutions at time *t*, while W*_SPOTPE_* is the weight of SPOTPE in the complex.

### 2.6. Characterizations

The ^1^H NMR measurements of TPEOH and SPOTPE in *d*_6_-DMSO were recorded on a 300 MHz spectrometer (Varian INOVA-300) at 25 °C. The chemical shifts were identified by the signals of tetramethylsilane (TMS). The ζ-Potentials of QC, SPOTPE, SPOTPE/QC and SPOTPE/QC+Fe^3+^ solutions were performed on a Nano-ZS ZEN3600 (Malvern Instruments, Worcestershire, UK) at 25 °C for three times to obtain the average values. The fluorescent spectra were measured on a spectrofluorophotometer (Perkin Elmer LS55) with an excitation wavelength at 336 nm and an emission wavelength at 472 nm. The excitation and emission slit widths were 10 nm and 4 nm, respectively. Photographs were obtained by a camera under a UV lamp with 365 nm excitation wavelength through the whole experiment.

Transmission electron microscopy (TEM) images were observed on a JEM-2100 (HR) electron microscope, using an accelerating voltage of 200 kV. TEM samples were prepared by dropping solutions onto copper grids coated with Formvar films, until the solvents were evaporated in a dust protected atmosphere. The dynamic laser light scattering (DLS) was performed on a light scattering goniometer (ALV/CGS-8F, ALV, Hessen, Germany) with a wavelength 632.8 nm from a He–Ne laser. The scattering angle (θ) was set at 90°. All of the sample solutions were filtered into the light-scattering bottles through the 0.45 μm filter (NYL, Whatman, UK). 

## 3. Results and Discussion

### 3.1. Physicochemical Properties of SPOTPE/QC Solutions

**Scheme 1:**
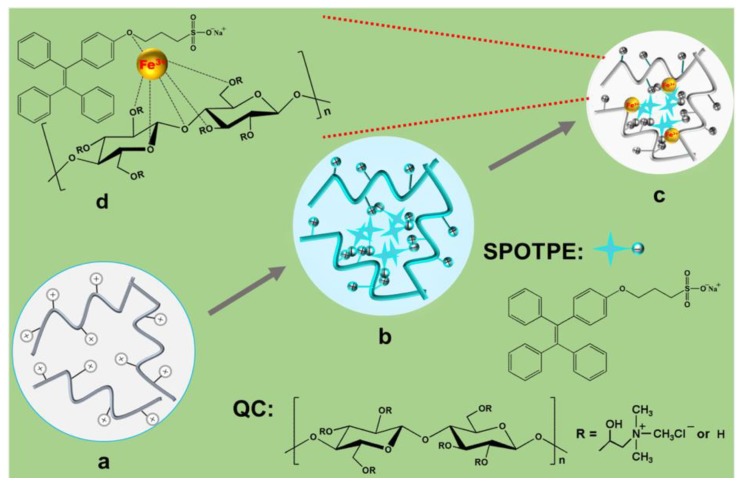
Preparation of 1,1,2-triphenyl-2-[4-(3-sulfonatopropoxyl)-phenyl]-ethene sodium salt (SPOTPE)/quaternized cellulose (QC) complexes for the detection of Fe^3+^.

**Figure 1:**
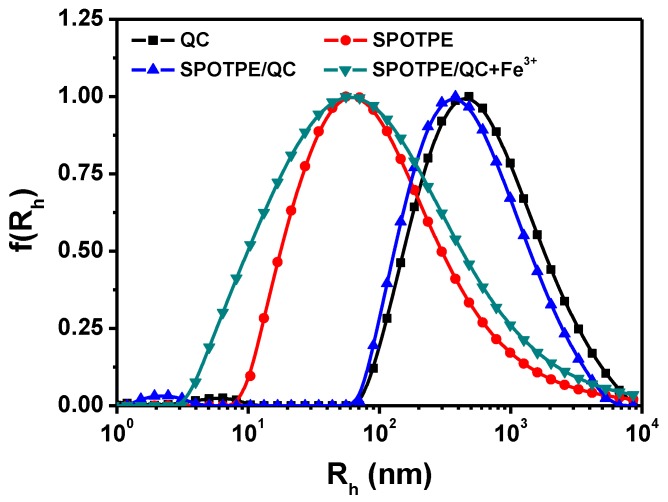
Hydrodynamic radius (R_h_) distributions of QC, SPOTPE, and SPOTPE/QC with/without Fe^3+^ in aqueous solutions determined by dynamic laser light scattering (DLS).

QC is a typical cationic polymer, which displays good solubility in water attributed to the presence of -(CH_3_)_3_N^+^ groups. As shown in Figure 1 and Table 1, the hydrodynamic radius (R_h_) and ζ-potential of QC in aqueous solutions were determined to be 571 nm and 48.3 mV, respectively. SPOTPE could be dissolved not only in common organic solvents, but also in polar solvents including water. The R_h_ and ζ-potential values of SPOTPE in water were determined to be 82 nm and −14.6 mV, respectively. As shown in Scheme 1, QC and SPOTPE formed complexes through the electrostatic interactions between -SO_3_^−^ and -(CH_3_)_3_N^+^ groups. The R_h_ and ζ-potential values of SPOTPE/QC in aqueous solutions were determined to be 467 nm and 46.4 mV, respectively. The R_h_ value of SPOTPE/QC was smaller than that of QC which can attributed to the strong electrostatic interaction between QC and SPOTPE. However, the ζ-potential of QC hardly changed after the introduction of SPOTPE, suggesting that SPOTPE molecules were firmly embedded in the inner of the complexes. 

**Figure 2:**
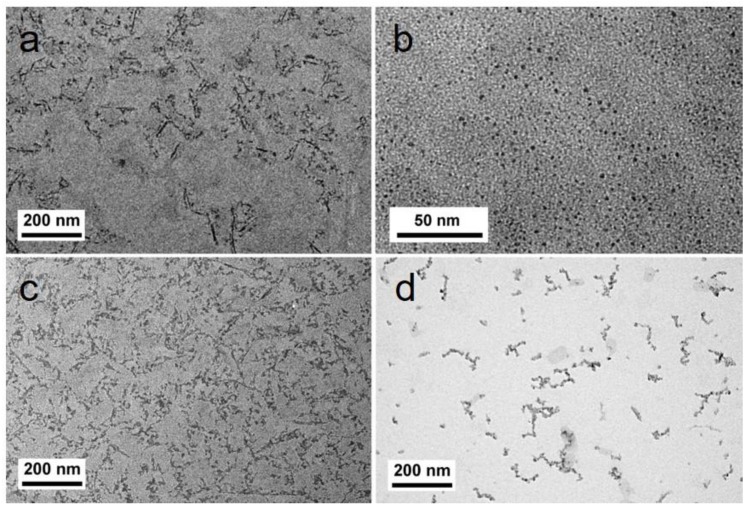
TEM images of (**a**) QC, (**b**) SPOTPE, (**c**) SPOTPE/QC, and (**d**) SPOTPE/QC+Fe^3+^.

Figure 2 shows the TEM images of QC, SPOTPE and SPOTPE/QC complexes. QC formed irregular nanowires in water due to the stiffness of the cellulose backbone and the electrostatic repulsion of the cationic polymer chains [37,38]. The average length of the QC nanowires was about 100 nm. For the strong hydrophobicity of the tetraphenylethylene structure, SPOTPE formed aggregated nanoparticles with a diameter between 2 nm and 5 nm. When the SPOTPE molecules were captured by QC chains through the electrostatic attraction, SPOTPE/QC complexes displayed a nanowire-shape as QC but with a smaller size. The length of the complexes was decreased to be about 80 nm, which was consistent with the results from DLS measurements (Table 1).

### 3.2. Fluorescence Properties of SPOTPE/QC Solutions

**Figure 3:**
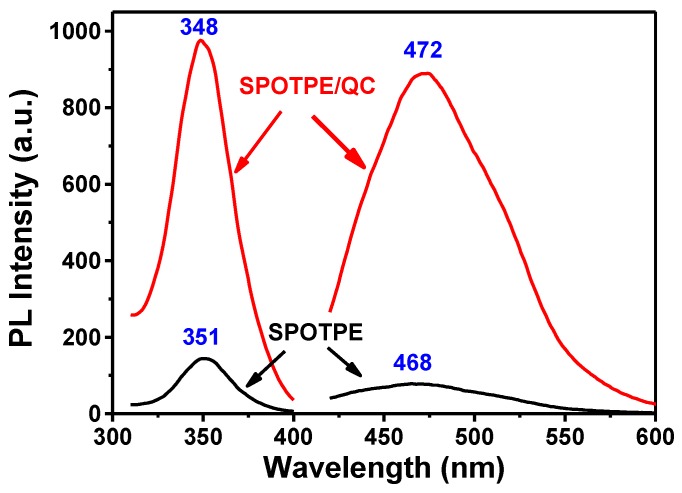
Fluorescence excitation and emission spectra of SPOTPE (0.2 mg/mL) and SPOTPE (0.01 mg/mL)/QC (0.2 mg/mL) solutions.

Figure 3 shows the fluorescent excitation and emission spectra of SPOTPE and SPOTPE/QC solutions. The SPOTPE solution (0.2 mg/mL) displayed a very weak fluorescence, and the maximum excitation and emission wavelengths were 351 nm and 468 nm, respectively. Interestingly, after introducing SPOTPE into the QC solutions, the fluorescence of SPOTPE (0.01 mg/mL)/QC (0.2 mg/mL) solution was remarkably enhanced with almost no change of the maximum excitation and emission wavelengths (348 nm and 472 nm, respectively). The QC solution showed no fluorescence (Figure S2), and the fluorescence of the SPOTPE/QC solution was just contributed by the aggregation of SPOTPE molecules. As shown in Scheme 1b, SPOTPE molecules aggregated in the QC nanowires due to the electrostatic interactions of their opposite charges. The aggregation of SPOTPE resulted in restricting the intramolecular rotation behavior of phenyl groups, leading to the increased radiative decay of the excitons, and showing enhanced fluorescence [13]. 

**Figure 4:**
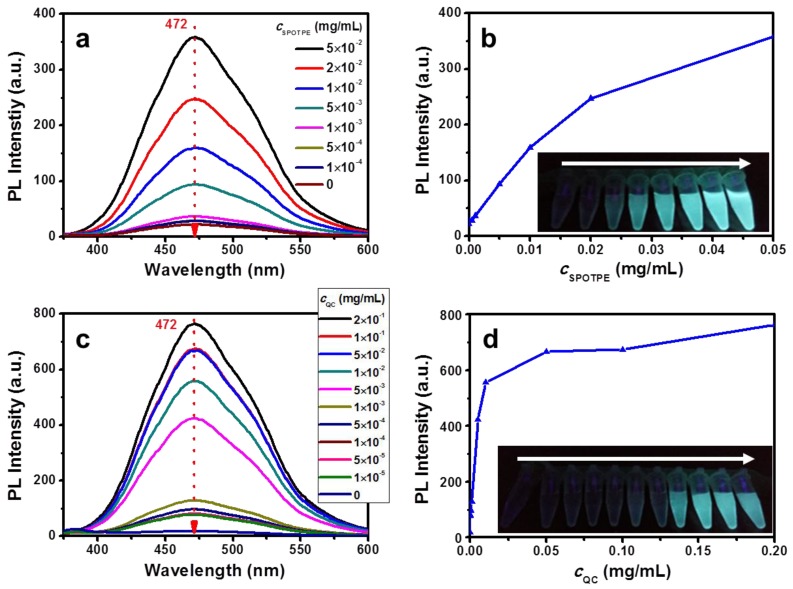
Fluorescence spectra of SPOTPE/QC solutions with various (**a**) SPOTPE (*c*_QC_ = 0.2 mg/mL) and (**c**) QC (*c*_SPOTPE_ = 0.01 mg/mL) concentrations. Dependence of the fluorescence intensity of SPOTPE/QC solutions at 472 nm on the (**b**) SPOTPE and (**d**) QC concentrations, insets were the photographs of the corresponding SPOTPE/QC solutions under the UV lamp (365 nm).

Figure 4 shows the fluorescent emission spectra of SPOTPE/QC solutions with various concentrations of SPOTPE or QC. Herein, the excitation and emission slit widths were 10 and 2.5 nm in Figure 4a, and were 10 and 4 nm in Figure 4c, respectively. As the *c*_QC_ was 0.2 mg/mL, the fluorescence intensity of the solutions increased with an increase of *c*_SPOTPE_, which displayed a typical AIE behavior (Figure 4b). Inset of Figure 4b was the picture of the QC solutions with increasing *c*_SPOTPE_. The solutions changed from colorless to strong cyan under irradiation from a hand-held UV lamp (365 nm). Interestingly, as the *c*_SPOTPE_ fixed at 0.01 mg/mL, the fluorescence intensity of the solutions dramatically increased as the *c*_QC_ increased from 0 mg/mL to 0.02 mg/mL, and then slightly increased with further increasing of *c*_QC_ from 0.02 mg/mL to 0.2 mg/mL (Figure 4d). A gradual increase of cyan fluorescence could also be observed by the naked eye (inset of Figure 4d). These phenomena suggested that the negatively charged SPOTPE molecules were aggregated in the positively charged QC chains, resulting in the florescence of the solutions.

**Figure 5:**
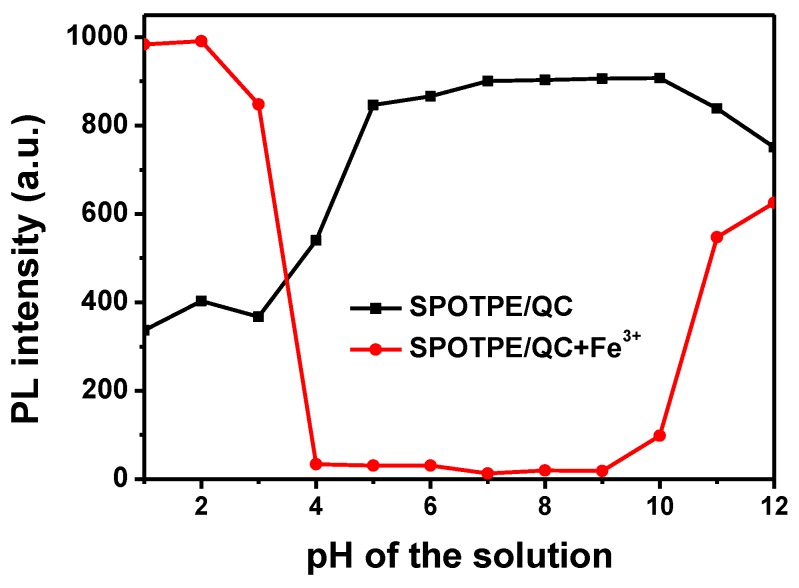
Dependence of the fluorescence intensity at 472 nm on the pH of the SPOTPE/QC solutions with/without Fe^3+^ (*c*_QC_ = 0.2 mg/mL, *c*_SPOTPE_ = 0.01 mg/mL, *c*_Fe_^3+^ = 300 μM).

The dependence of the fluorescence intensity at 472 nm on the pH of the SPOTPE/QC solutions is shown in Figure 5. SPOTPE/QC solutions displayed stable fluorescence over a pH range of 5.0–10.0. When the pH was lower than 5.0, the fluorescence of the solutions decreased sharply contributing to the disaggregation of SPOTPE. As the pH was higher than 10.0, the fluorescence of the solutions also decreased, because QC dissolved well in the alkaline solutions. On the other hand, the strong interaction between OH^−^ and -(CH_3_)_3_N^+^ groups hindered the combination of SPOTPE molecules with QC chains. 

### 3.3. Selective Detection of *Fe^3+^ Ions*

**Figure 6:**
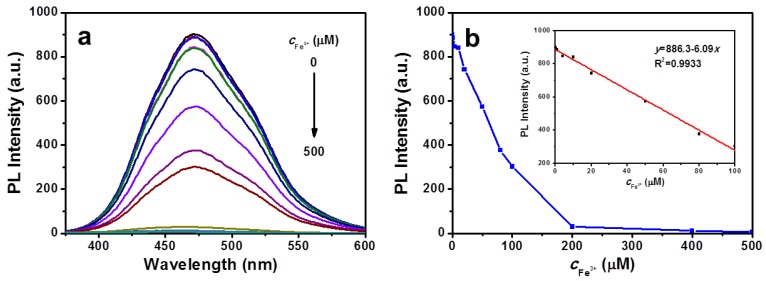
(**a**) Fluorescence spectra of SPOTPE/QC solutions with the addition of Fe^3+^. (**b**) Dependence of the fluorescence intensity of SPOTPE/QC solutions at 472 nm on the Fe^3+^ concentrations. (*c*_QC_ = 0.2 mg/mL, *c*_SPOTPE_ = 0.01 mg/mL).

The fluorescence emission spectra and fluorescence intensity at 472 nm of SPOTPE/QC solutions with the addition of Fe^3+^ are shown in Figure 6. The fluorescence decreased sharply with the concentration of Fe^3+^ increasing over 0–200 μM. Linear relationship between the fluorescence intensity and Fe^3+^ concentration was observed in the range of 0 μM−100 μM (Inset of Figure 6b). The detection limit of SPOTPE/QC solution for Fe^3+^ was determined to be 2.92 × 10^−6^ M according to the equation: LOD = 3S/σ (While S is the standard deviation of the blank samples for 10 times, σ is the slope of the line mentioned in Figure 6b inset) [39]. The fluorescence quenching efficiency of SPOTPE/QC solution was 98% according to the equation: *ϕ* = (*F*_0_ − *F*)/*F*_0_, where *F*_0_ was the fluorescence intensity of SPOTPE/QC, and *F* was the fluorescence intensity of SPOTPE/QC+Fe^3+^ (Figure S2). Moreover, the quenching process occurred quickly. When injecting 9 mM Fe^3+^ ions into the SPOTPE/QC solution, the fluorescent intensity was quenched from 907 to 3 within 5 s (Figure S3). SPOTPE/QC solution demonstrated a sensitive detection for Fe^3+^ ions at a very low SPOTPE concentration, because QC significantly amplified the AIE effect of SPOTPE. 

Figure 5 also shows the dependence of the fluorescence intensity on pH of the SPOTPE/QC solutions with the addition of Fe^3+^ ions (300 μM). It is shown that the fluorescence of the solutions could be quenched by Fe^3+^ ions without the interference of H^+^ and OH^−^ in a pH range of 4.0–10.0. In a more alkaline solution (pH > 10), Fe^3+^ ions formed into Fe(OH)_3_ colloids. SPOTPE molecules aggregated with the colloids, resulting in an increase fluorescence of the solutions. In the acidic media, QC displayed poor solubility and formed large aggregates, which benefited the flocculation of SPOTPE molecules, and also resulted in increasing the fluorescence of the solutions. 

**Figure 7:**
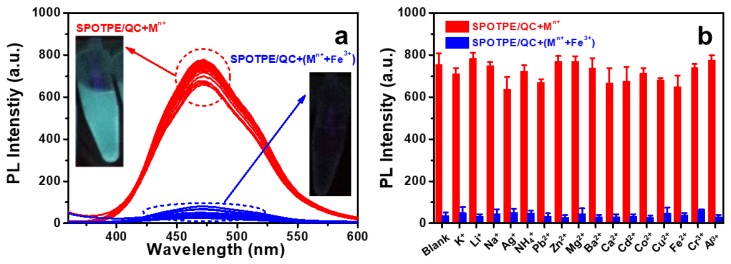
(**a**) Fluorescence emission spectra and (**b**) fluorescence intensity at 472 nm of SPOTPE/QC+M^n+^ solutions with/without Fe^3+^. Insets were the photographs of SPOTPE/QC+M^n+^ solutions with/without Fe^3+^ under the UV lamp (365 nm). (M^n+^: K^+^, Li^+^, Na^+^, Ag^+^, NH_4_^+^, Pb^2+^, Zn^2+^, Mg^2+^, Ba^2+^, Ca^2+^, Cd^2+^, Co^2+^, Cu^2+^, Fe^2+^, Cr^3+^ or Al^3+^) (*c*_QC_ = 0.2 mg/mL, *c*_SPOTPE_ = 0.01 mg/mL, *c*_M_^n+^ = 300 μM, *c*_Fe_^3+^ = 300 μM).

Interfering ions (M^n+^: K^+^, Li^+^, Na^+^, Ag^+^, NH_4_^+^, Pb^2+^, Zn^2+^, Mg^2+^, Ba^2+^, Ca^2+^, Cd^2+^, Co^2+^, Cu^2+^, Fe^2+^, Cr^3+^ or Al^3+^) were chosen to investigate the selective detection of SPOTPE/QC solution. As shown in Figure 7, SPOTPE/QC solutions displayed strong fluorescence in the presence of the interfering ions. With the addition of M^n+^+Fe^3+^ ions, the fluorescence of the solutions quenched quickly. The results clearly illustrated that the SPOTPE/QC solution performed a selective detection towards Fe^3+^ ions against the interfering ions, including monovalent, divalent and trivalent ions. The competitive ions had no effects on the Fe^3+^ detection assay. Moreover, the apparent changes in fluorescence could also be observed by the naked eye (inset of Figure 7a). 

### 3.4. Quenching Mechanism

**Figure 8:**
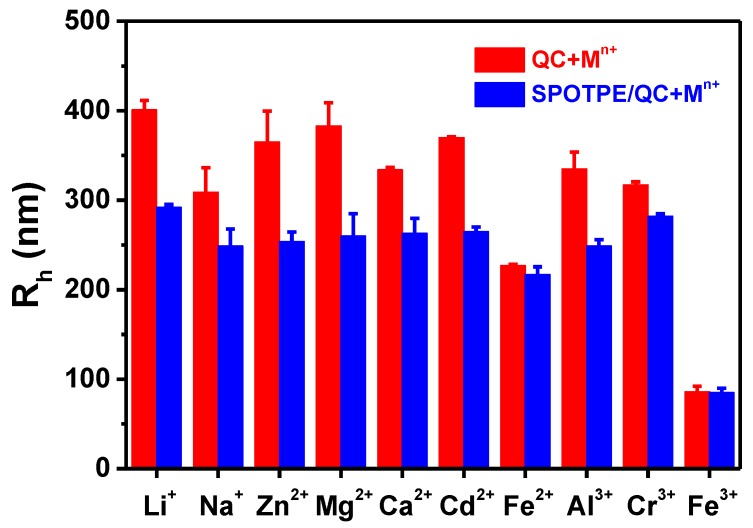
Hydrodynamic radius (R_h_) of QC+M^n+^ and SPOTPE/QC+M^n+^ (M^n+^: Li^+^, Na^+^, Zn^2+^, Mg^2+^, Ca^2+^, Cd^2+^, Fe^2+^, Al^3+^, Cr^3+^ or Fe^3+^) determined by DLS in aqueous solutions.

Figure 8 shows the R_h_ values of QC+M^n+^ and SPOTPE/QC+M^n+^ in aqueous solutions. The R_h_ values of QC+M^n+^ were in the range of 300–400 nm, which were smaller than that of QC (571 nm) because the metal ions could flocculate with QC. The R_h_ values of SPOTPE/QC+M^n+^ were in the range of 200–300 nm, which were smaller than those of SPOTPE/QC and QC+M^n+^. This can be attributed to the introduction of the negatively charged SPOTPE. Interestingly, the R_h_ values of QC+Fe^3+^ and SPOTPE/QC+Fe^3+^ were 86 nm and 85 nm, respectively, which were much smaller than those of QC+M^n+^ and SPOTPE/QC+M^n+^ systems. The results indicated that Fe^3+^ could form much more compact composites with QC than the other metal ions. TEM image of Figure 2d also confirmed that SPOTPE/QC+Fe^3+^ formed compact nanowires in the aqueous solutions. Figure S4 shows the release profiles of SPOTPE from the SPOTPE/QC complexes. The cumulative release of SPOTPE from the complexes in the presence/absence of Fe^3+^ ions was only 25% and 32%, respectively, which further suggested a strong electrostatic interaction between SPOTPE and QC. Moreover, the ζ-potential of SPOTPE/QC+Fe^3+^ was 42.7 mV, which was lower than that of SPOTPE/QC (46.4 mV). All of the results suggested that Fe^3+^ ions strongly interacted with SPOTPE and QC, and Fe^3+^ and SPOTPE were firmly embedded inside the complex.

On the other hand, Fe^3+^ has paramagnetic properties and an unfilled *d* shell, so that it has the propensity to quench the fluorescence of the neighboring fluorophore by energy or electron transfer (Figure S5) [40,41]. Therefore, the quenching mechanism could be described from two aspects. One is the strong interactions among Fe^3+^ ions, SPOTPE molecules and QC chains, which make the distance between Fe^3+^ and SPOTPE close enough. The other is the electron/energy transfer occurring between Fe^3+^ and SPOTPE. As illustrated in Scheme 1d, a hypothesis was proposed as the ether groups on the SPOTPE and QC chain could coordinate with Fe^3+^ ions to form a complex, which allowed a non-radiative deactivation and quenched the fluorescence of SPOTPE. As a result, SPOTPE/QC solutions achieved the selective and sensitive detection for Fe^3+^ ions.

## 4. Conclusions

In summary, a SPOTPE/QC fluorescent complex was successfully constructed for the detection of Fe^3+^ ions in aqueous solutions. SPOTPE molecules were firmly embedded in the SPOTPE/QC complex through the strong electrostatic interactions between -SO_3_^−^ and -(CH_3_)_3_N^+^ groups. The AIE effect of SPOTPE was significantly amplified after introducing QC into the SPOTPE aqueous solutions. The SPOTPE/QC solutions displayed stable fluorescence over a wide pH range of 5.0–10.0. The SPOTPE/QC solutions demonstrated a selective and sensitive response to Fe^3+^ ions with ignored interferences of other ions, and the detection limit was determined to be 2.92 × 10^−6^ M. A hypothesis of the quenching mechanism was ascribed to the close proximity and electron/energy transfer between Fe^3+^ ions and the excited SPOTPE molecules. This polymeric complex demonstrated a rapid and selective fluorescence detection over a wide pH range, and it has a promising application in the detection of Fe^3+^ ions in aqueous solutions.

## Figures and Tables

**Table 1:** Parameters of QC, SPOTPE, SPOTPE/QC and SPOTPE/QC+Fe^3+^ in aqueous solution.

Code	QC	SPOTPE	SPOTPE/QC	SPOTPE/QC+Fe^3+^
ζ (mV)	48.3	−14.6	46.4	42.7
R_h_ (nm)	571	82	467	85

## Supplementary Materials

The following are available online at http://www.mdpi.com/2079-4991/9/2/279/s1, Scheme S1: Synthesis of SPOTPE, Figure S1: ^1^H NMR spectra of TPEOH and SPOTPE in *d*_6_-DMSO, Figure S2: Fluorescence emission spectra of QC, SPOTPE/QC, and SPOTPE/QC+Fe^3+^ aqueous solutions (*c*_QC_ = 0.2 mg/mL, *c*_SPOTPE_ = 0.01 mg/mL, *c*_Fe_^3+^ = 300 μM), Figure S3: Fluorescence intensity kinetics of SPOTPE/QC solution with the addition of Fe^3+^ ions (*c*_QC_ = 0.2 mg/mL, *c*_SPOTPE_ = 0.01 mg/mL, *c*_Fe_^3+^ = 9 mM), Figure S4: In vitro SPOTPE-released profiles of SPOTPE/QC complex with/without Fe^3+^ ions (300 μM) in water, Figure S5: Fluorescence emission spectra of Fe^3+^, SPOTPE, and SPOTPE+Fe^3+^ aqueous solutions (*c*_SPOTPE_ = 0.2 mg/mL, *c*_Fe_^3+^ = 300 μM).
