# Supplementary material for: Self-Assembly Fluorescent Cationic Cellulose Nanocomplex via Electrostatic Interaction for the Detection of Fe3+ Ions
Source: Nanomaterials (Basel). 2019 Feb 16;9(2):279. doi: 10.3390/nano9020279 (PMC6410059; doi:10.3390/nano9020279)
Supplement: Supplementary file 1 [file nanomaterials-09-00279-s001.pdf]

*Supplementary Materials*

# Self-Assembly Cationic Cellulose Nanocomplex via Electrostatic Interaction for the Fluorescent Detection of $Fe^{3+}$ Ions

**Haoying Wang, Xiu Ye and Jinping Zhou***

Department of Chemistry, Wuhan University, Wuhan 430072, Hubei, China; wang159hy@gmail.com (H.W.); yx444131997@gmail.com (X.Y.)
*Correspondence: zhoujp325@whu.edu.cn; Tel.: +86-27-68752977 (J.Z)

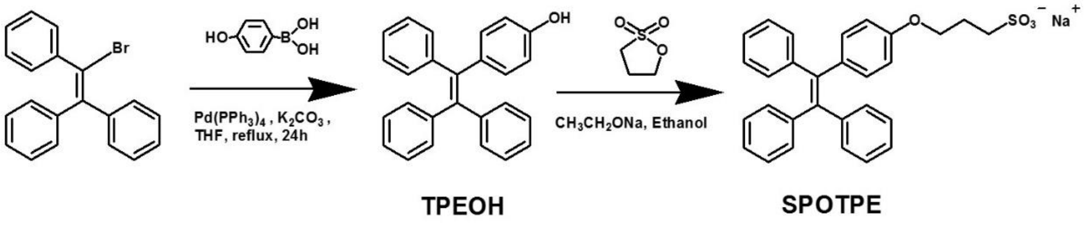


**Scheme S1.** Synthesis of SPOTPE.

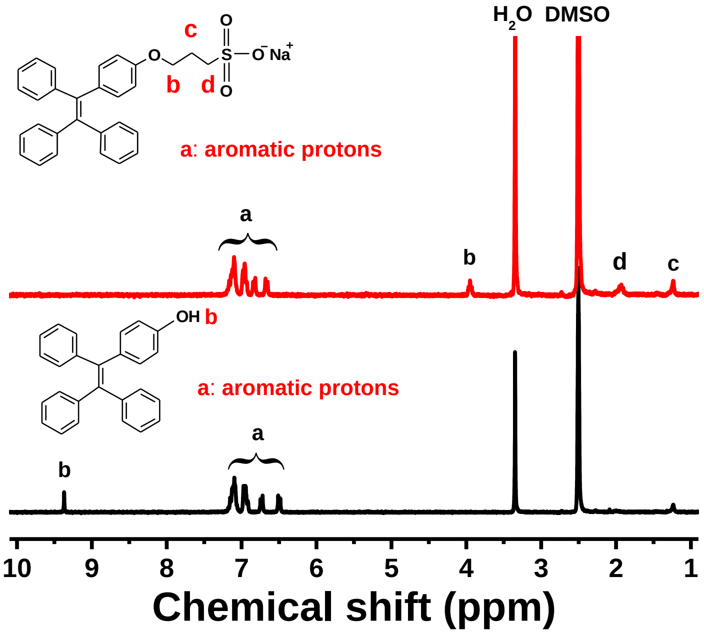


**Figure S1.** $^1H$ NMR spectra of TPEOH and SPOTPE in DMSO-$d_6$ at 25 °C.

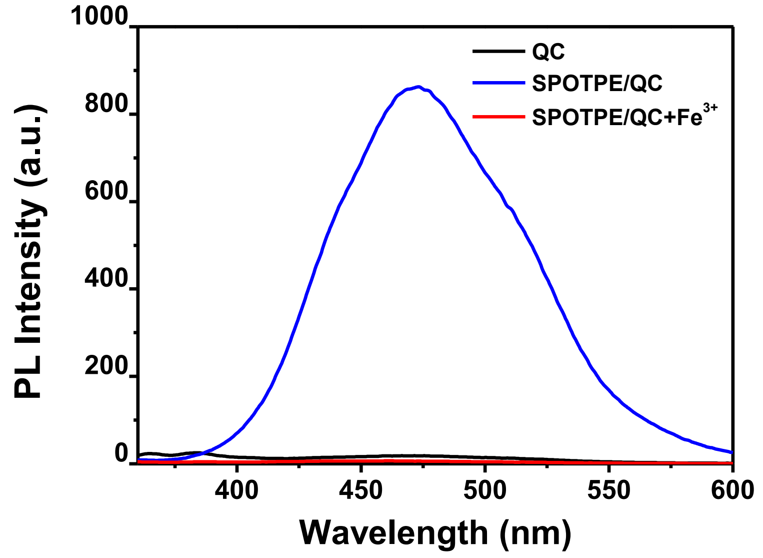


**Figure S2.** Fluorescence emission spectra of QC, SPOTPE/QC, and SPOTPE/QC+$Fe^{3+}$ aqueous solutions ($c_{QC}$=0.2 mg/mL, $c_{SPOTPE}$=0.01 mg/mL, $c_{Fe^{3+}}$=300 μM).

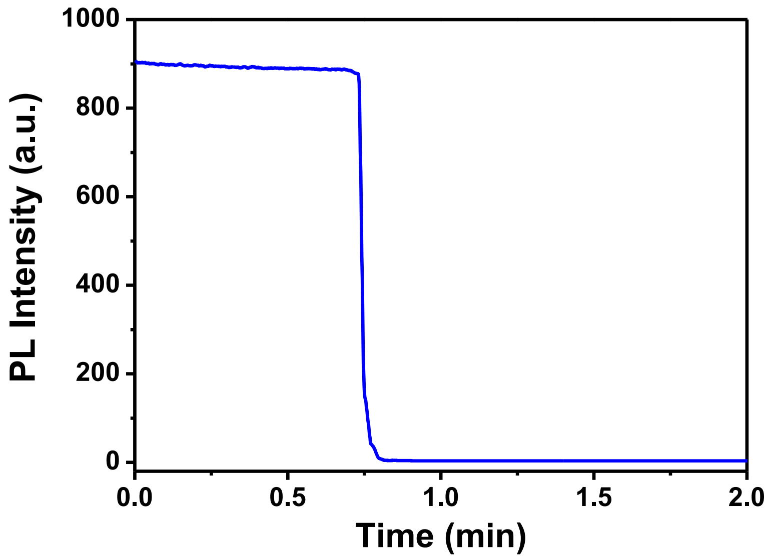


**Figure S3.** Fluorescence intensity kinetics of SPOTPE/QC solution with the addition of $Fe^{3+}$ ions ($c_{QC}$=0.2 mg/mL, $c_{SPOTPE}$=0.01 mg/mL, $c_{Fe^{3+}}$=9 mM).

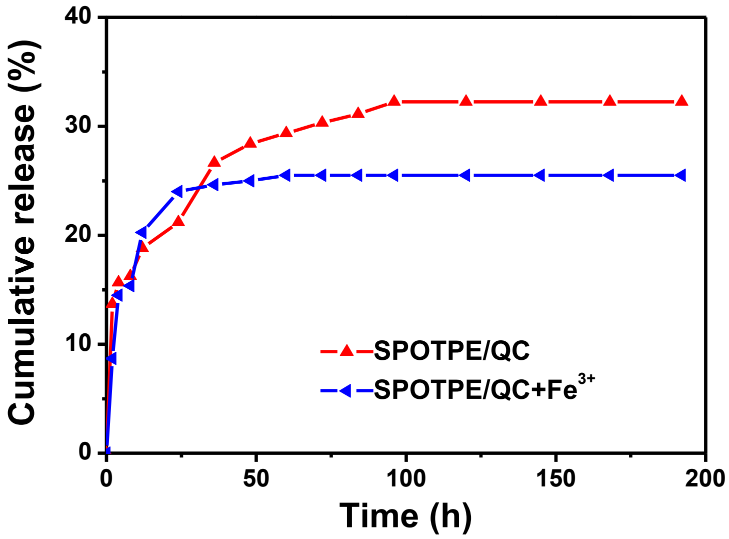


**Figure S4.** In vitro SPOTPE-released profiles of SPOTPE/QC complex with/without $Fe^{3+}$ ions (300 μM) in water.

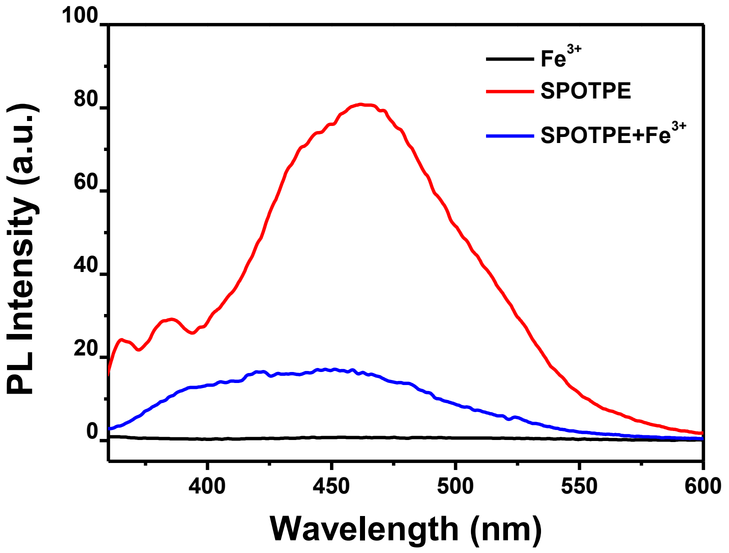


**Figure S5.** Fluorescence emission spectra of $Fe^{3+}$, SPOTPE, and SPOTPE+$Fe^{3+}$ aqueous solutions ($c_{SPOTPE}$=0.2 mg/mL, $c_{Fe^{3+}}$=300 μM).
